# Optical Microbubble Resonators with High Refractive Index Inner Coating for Bio-Sensing Applications: An Analytical Approach

**DOI:** 10.3390/s16121992

**Published:** 2016-11-25

**Authors:** Andrea Barucci, Simone Berneschi, Ambra Giannetti, Francesco Baldini, Alessandro Cosci, Stefano Pelli, Daniele Farnesi, Giancarlo C. Righini, Silvia Soria, Gualtiero Nunzi Conti

**Affiliations:** 1 Istituto di Fisica Applicata Nello Carrara (CNR IFAC), Via Madonna del Piano 10, 50019 Sesto Fiorentino, Firenze, Italy; a.barucci@ifac.cnr.it (A.B.); s.berneschi@ifac.cnr.it (S.B.); a.giannetti@ifac.cnr.it (A.G.); f.baldini@ifac.cnr.it (F.B.); a.cosci@ifac.cnr.it (A.C.); s.pelli@ifac.cnr.it (S.P.); d.farnesi@ifac.cnr.it (D.F.); giancarlo.righini@centrofermi.it (G.C.R.); g.nunziconti@ifac.cnr.it (G.N.C.); 2 Centro Studi e Ricerche «E. Fermi» Piazza del Viminale 1, 00184 Rome, Italy

**Keywords:** whispering gallery mode resonators, optical microbubble resonator, optical modeling, high refractive index polymer

## Abstract

The design of Whispering Gallery Mode Resonators (WGMRs) used as an optical transducer for biosensing represents the first and crucial step towards the optimization of the final device performance in terms of sensitivity and Limit of Detection (LoD). Here, we propose an analytical method for the design of an optical microbubble resonator (OMBR)-based biosensor. In order to enhance the OMBR sensing performance, we consider a polymeric layer of high refractive index as an inner coating for the OMBR. The effect of this layer and other optical/geometrical parameters on the mode field distribution, sensitivity and LoD of the OMBR is assessed and discussed, both for transverse electric (TE) and transverse magnetic (TM) polarization. The obtained results do provide physical insights for the development of OMBR-based biosensor.

## 1. Introduction

Nowadays, it is well established how the design of Whispering Gallery Mode Resonators (WGMRs) used as transducers for sensing and/or biosensing applications represent the first and crucial step towards the optimization of the final device performance [1]. Among all the different WGMRs typologies reported in literature and concerning this topic, such as microsphere [2,3], ring resonator based on bulk cylindrical filament or fiber [4,5,6], microdisk [7,8], and microtoroids [9,10], the hollow core WGM microcavities present the great advantage of having an embedded microfluidics, which makes them a promising platform for the development of high performance integrated sensors and/or biosensors. The liquid core optical ring resonators (LCORR) were the first hollow WGMR [11,12,13,14,15,16]. In order to enhance the LCORR sensitivity, different strategies were studied and proposed such as the decrease of the microcavity shell thickness [17], the addition of a polymeric coating of the LCORR inner surface [18,19] and the excitation of high order modes [20,21].

On the other hand, Lane and coworkers theoretically and experimentally proved high sensing performance, both as refractometer and biosensor, for silica microcapillaries coated with a thin layer of silicon quantum dots [22,23,24]. As an alternative, Rowland and coauthors considered the case of a sub-wavelength, high refractive index polymeric layer, doped with a dye, as a fluorescent coating for the inner side of thick-walled silica capillaries [25].

Optical microbubble resonators (OMBR) present a localized hollow spherical section with larger radial size respect to those cylindrical ones of the overall device. In order to induce the aforementioned localized swelling in a dielectric cylindrical microtube, different fabrication processes have been developed. Each of them considers a local heating of a fused silica microcapillary by means of a CO_2_ laser or an arc discharge while, the hollow core microcylinder is internally pressurized contemporarily. Adjusting the fabrication parameters, a good control on the OMBR size and shell thickness may be reached [26,27,28]. From an electromagnetic point of view, the greater curvature of the OMBRs around the equatorial plane improves the light confinement along the polar direction and guarantees higher Q factor values in comparison with the previous LCORR devices, even when the wall thickness is in the sub-wavelength range [29]. Moreover, it was theoretically demonstrated how these novel class of hollow core WGMRs have a sensing capability well greater than those presented by the corresponding LCORRs and bulk microspheres, depending on the shell thickness value. This feature, joined with their naturally integrated capillary-based microfluidics, makes them a very promising platform for sensing application [30].

As reported for the LCORR, the most commonly used method to extend the WGM evanescent field in the sensing region of an OMBR (i.e., its inner volume or surface, depending on the type of application) and thus increase the device performance is given by a drastic decrease of the microbubble wall thickness during the fabrication process, in a way that the shell of the final device so obtained starts to lose its capability to confine the WGMs within itself. This represents the so-called “quasi-droplet” condition and, generally, it is associated with wall thickness values comparable with the excitation wavelength or lower. The achievement of this goal requires a strong control on the fabrication parameters and makes the OMBR extremely fragile from a mechanical point of view [31,32].

In this paper, we propose an analytical method for the design of an OMBR-based biosensor that enhances the sensitivity or the fraction of the field intensity in the biological layer. We consider the presence of a high refractive index polymeric coating on the OMBR inner surface and we theoretically investigate the effect of this layer on the mode field distribution and device performance, both for TE and TM polarization. The purpose of this work is twofold: on the one hand, demonstrate that the presence of this high refractive index layer enhances the OMBR sensitivity as biosensor such as in a microsphere [33,34,35,36]; and, on the other hand, prove that the characteristic of having a sub-wavelength wall thickness for the OMBR does not always represent a requirement for the achievement of high device performance. This allows having mechanically robust hollow core microcavities without losing in sensitivity or limit of detection (LoD). Regarding the polymer, our choice has fallen on SU-8 for its excellent mechanical and chemical stability, which makes it a successful material in many application fields, from microfluidics to communications and sensing [37]. Moreover, its refractive index value is one of the highest among all the polymeric materials, well higher than both silica and the liquid core, which is typically water or a buffer solution (PBS) in biosensing applications. The excitation wavelength, selected for our study, is 780 nm, as water absorption at this wavelength is relatively low (~10^−2^ cm^−1^, about three orders of magnitude lower than at 1.55 μm). The modeling is performed in order to optimize the main geometrical parameters of the system such as the microbubble size and wall thickness, and the inner coating thickness, taking also into account ideal phase matching condition for the excited WGM order. The obtained results may provide physical insights for the development of OMBR-based biosensor.

## 2. Methods 

### 2.1. OMBR Analytical Model

**Figure 1:**
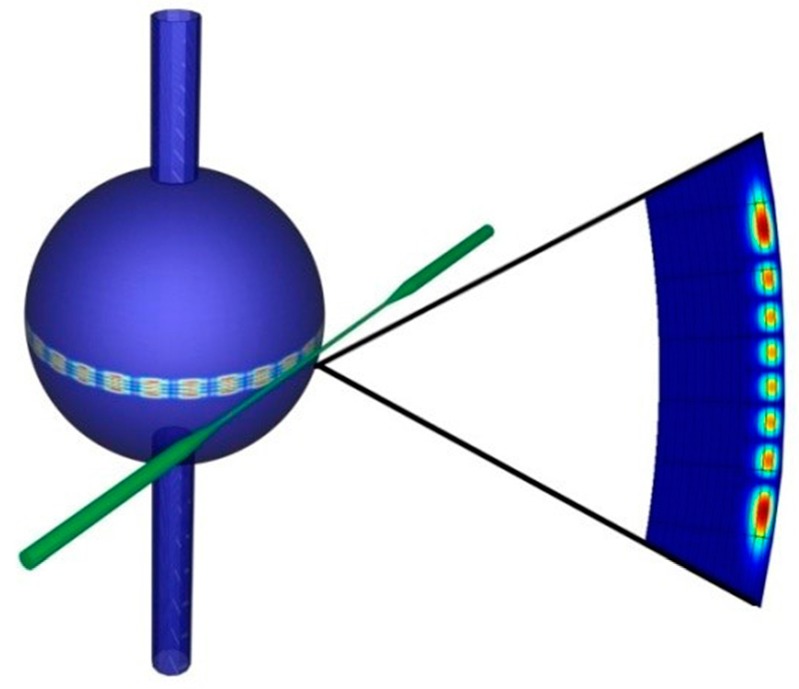
Three dimensional render image of an OMBR coupled with a fiber taper. An example of field intensity cross section on the hollow WGM microcavity surface is reported for *n* = 1 and *l* − |*m*| + 1 = 8.

Figure 1 represents a sketch of an OMBR where the bubble section assumes a spherical shape “embedded” in two cylindrical capillary sections that represent the microfluidic ports of the overall system. The WGMs are excited by the evanescent field of the mode propagating in the excitation system (i.e., a guided structure as a tapered fiber) and travel glancing to the resonator surface by total internal reflection (TIR) all along the equatorial plane [2,30]. Due to the fact that the physical structure of a microbubble is similar to that of a microsphere (i.e., in effect, a microsphere can be viewed as a microbubble with the inner medium made of the same material as the shell), one could foresee that the WGMs spatial distribution continues to be similar in both optical resonant microsystems. This is generally true, but with some slight differences. In fact, conversely from a microsphere, the WGMs resonance spectrum in an OMBR is determined not only by the microresonator size and the refractive index of the outer medium but also depends on the spatial distribution of the refractive index inside the hollow microcavity. Moreover, if the OMBR shell thickness is comparable with the excitation wavelength (i.e., “quasi-droplet” regime) or the OMBR is filled with some fluid, the radial component of the electromagnetic field changes its distribution in comparison with that supported by a microsphere having the same size [27]. However, due to the spherical symmetry, the WGMs in an OMBR can be identified by three different mode number *l, m* and *n* as defined in the microsphere case [2]:

- *l* is the azimuthal mode number and it is linked to the max.–min. number of the periodical functions sin(x) and cos(x) along the equatorial plane;
- *m* is the polar mode number and it is related to the max.−min. number of the harmonic Legendre functions in the polar direction (n° max. in the polar direction: *l* − *m* + 1); and
- *n* is associated to the max.–min. number of the field in the radial direction.

For an ideal sphere, modes with the same *l* and *n*, but arbitrary *m*, have the same resonant wavelength. When *m* = *l* and *n* = 1, the modes are called fundamental WGMs. Moreover, each WGM is also characterized by its polarization: Transverse Electric (TE) where the electric field is parallel to the surface, or Transverse Magnetic (TM) where the electric field is perpendicular to the surface.

Applying the appropriate boundary conditions leads to the equations for the TE and TM polarizations, which have an infinite number of roots, corresponding to different radial mode orders. 

**Figure 2:**
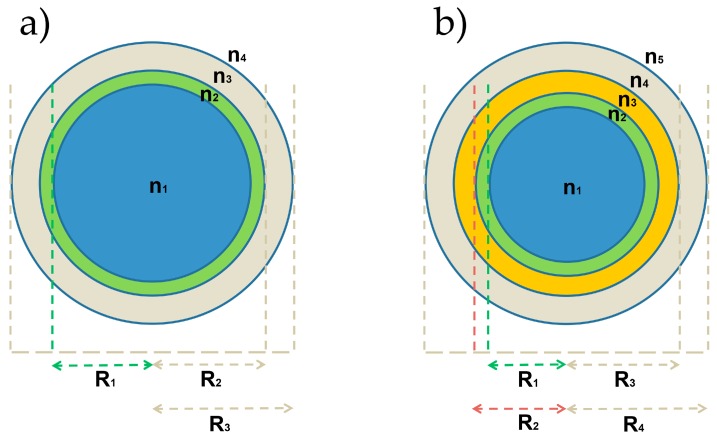
Cross sections of an OMBR-based biosensor: (**a**) 4-layer structure, where there is only a biological layer on the OMBR inner surface; and (**b**) 5-layer structure, where between the biological layer and the OMBR inner surface there is a high refractive index polymeric layer (SU-8).

We investigated here two different configurations for our OMBR-based biosensor, with or without a high refractive index polymeric layer. The former case is defined as a 4-layer structure, which consists of a liquid core (buffer solution, *n*_1_ = 1.33), a biological layer thin film (*n*_2_ = 1.46), the OMBR silica shell (*n*_3_ = 1.45) and the surrounding layer (air, *n*_4_ = 1). The latter case is related to a 5-layer structure, in which, between the biological layer (*n*_2_ = 1.46) and the OMBR inner surface (*n*_4_ = 1.45), there is an SU-8 polymer film (*n*_3_ = 1.58). Figure 2 shows a sketch of the two structures under investigation. The working wavelength is selected at 780 nm. The thickness of the biological layer has been set to 20 nm, in agreement with the reported values in literature [38,39]. The index of refraction has been chosen as a value in between the index of refraction of a dried protein (~1.55) and the refractive index of the solvent (~1.36).

It is worth noting as the mathematical formalism associated to the physical problem is independent on the number of layers involved, so both our coating conditions can be investigated just using the right number of equations describing each layer. In fact, any layer corresponds to an equation and consequently it involves two boundary conditions. Assuming a microbubble as a hollow microsphere with a given shell thickness, the electromagnetic problem can be solved in spherical coordinates represented by the usual variables: *r* for the radial direction, *θ* for the polar direction and *φ* for the azimuthal one. Due to the orthonormality of the variables and under the assumption that the direction of polarization associated with the electromagnetic field can be considered constant through all points in space of a spherical coordinates system, the Helmholtz equation is separable and the corresponding field solution can be expressed in the form: Ψ*_l,m,n_*(*r*, *θ*, *φ*) = N *P_r_*(*r*) *Y_l,m_*(*θ*, *φ*), where N is the normalization constant, and *P_r_*(*r*) and *Y_l,m_*(*θ*, *φ*) are the radial and the angular contributions of the field, respectively, where *P_r_*(*r*) = *S_r_*(*r*) for the TE mode and *P_r_*(*r*) = *T_r_*(*r*) for the TM mode. The *Y_l,m_*(*θ*, *φ*) contribution, which includes in its inside information on the polar and azimuthal field components, is a function both of the spherical harmonics, with *l*th degree and *m*th order, and the periodical functions sin(*m*φ) and cos(*m*φ) [18,33,34,35]. For an OMBR with 5-layer structure, as reported in Figure 2b, the radial component *S_r_*(*r*) for the TE mode can be written as:

(1) $$S_{r}(r)=\{\begin{array}{c} a_{1}j_{l}(kn_{1}r),r\leq R_{1}\\ a_{2}j_{l}(kn_{2}r)+b_{2}h_{l}^{1}(kn_{2}r)R_{1}\leq r\leq R_{2}\\ a_{3}j_{l}(kn_{3}r)+b_{3}h_{l}^{1}(kn_{3}r)R_{2}\leq r\leq R_{3}\\ a_{4}j_{l}(kn_{4}r)+b_{4}h_{l}^{1}(kn_{4}r)R_{3}\leq r\leq R_{4}\\ a_{5}j_{l}(kn_{5}r),r\leq R_{4} \end{array}$$

where *k* is the resonant wave vector; *R*_1_ is the distance between the OMBR center and the biological layer; *R*_2_ is the distance from the OMBR center to the polymeric layer; *R*_3_ the OMBR inner radius; *R*_4_ is the OMBR outer radius; Δ*_bio_* = *R*_2_ − *R*_1_ is the biological layer thickness; Δ*_poly_* = *R*_3_ − *R*_2_ is the polymer thickness; and *w* = *R*_4_ − *R*_3_ is the OMBR wall thickness. The radial field behavior in the inner layer inside the OMBR can be described by the spherical Bessel function *j_j_*(*r*) while, for the outer layer, it can be associated to with first order spherical Hankel function *h_j_*^(*1*)^(*r*). In all the other layers of the structure, the radial field goes as a combination of the spherical Bessel function *j_j_*(*r*) and spherical Hankel function *h_j_*^(*1*)^(*r*). For the TM mode, the radial function *T_r_*(*r*) take a similar for to those of *S_r_*(*r*). The *a_i_* and *b_i_* coefficients, with *i* = 1, …, 5, are parameters that can be calculated analytically using the boundary conditions, which require that *S_r_*(*r*), *S*’*_r_*(*r*), *T_r_*(*r*) and *T*’*_r_*(*r*)/*n*^2^ be continuous across the interfaces:

(2) $$\left\{ \begin{array}{c} P_{r,i}(r){|}_{Ri}=P_{r,i+1}(r){|}_{Ri},P_{r,i}=S_{r,i}\;TE\;mode,P_{r,i}=T_{r,i}\;TM\;mode\\ \frac{\partial S_{r,i}}{\partial r}(r){|}_{Ri}=\frac{\partial S_{r,i+1}}{\partial r}(r){|}_{Ri},\;TE\;mode\\ \frac{1}{n_{i}^{2}}\frac{\partial T_{r,i}}{\partial r}(r){|}_{Ri}=\frac{1}{n_{i+1}^{2}}\frac{\partial T_{r,i+1}}{\partial r}(r){|}_{Ri}\;TM\;mode \end{array}\right.$$

The boundary conditions implementation at the different interfaces leads to a homogeneous linear system that can be written in matrix form as:

(3) $$M\left(\lambda ,l\right)\times \;\vec{A}=\vec{0}$$

where, for the TE mode, the matrix M(*λ*,*l*) can be represented as:
$j_{l}\left(kn_{1}R_{1}\right)$$-j_{l}\left(kn_{2}R_{1}\right)$$-h_{l}^{1}\left(kn_{2}R_{1}\right)$00000$n_{1}{j^{'}}_{l}\left(kn_{1}R_{1}\right)$$-n_{2}{j^{\prime }}_{l}\left(kn_{2}R_{1}\right)$$-n_{2}{h^{'}}_{l}^{1}\left(kn_{2}R_{1}\right)$000000$j_{l}\left(kn_{2}R_{2}\right)$$h_{l}^{1}\left(kn_{2}R_{2}\right)$$-j_{l}\left(kn_{3}R_{2}\right)$$-h_{l}^{1}\left(kn_{3}R_{2}\right)$0000$n_{2}{j^{'}}_{l}\left(kn_{2}R_{2}\right)$$n_{2}{h^{'}}_{l}^{1}\left(kn_{2}R_{2}\right)$$-n_{3}{j^{\prime }}_{l}\left(kn_{3}R_{2}\right)$$-n_{3}{h^{'}}_{l}^{1}\left(kn_{3}R_{2}\right)$000000$j_{l}\left(kn_{3}R_{3}\right)$$h_{l}^{1}\left(kn_{3}R_{3}\right)$$-j_{l}\left(kn_{4}R_{3}\right)$$-h_{l}^{1}\left(kn_{4}R_{3}\right)$0000$n_{3}{j^{'}}_{l}\left(kn_{3}R_{3}\right)$$n_{3}{h^{'}}_{l}^{1}\left(kn_{3}R_{3}\right)$$-n_{4}{j^{'}}_{l}\left(kn_{4}R_{3}\right)$$-n_{4}{h^{'}}_{l}^{1}\left(kn_{4}R_{3}\right)$000000$j_{l}\left(kn_{4}R_{4}\right)$$h_{l}^{1}\left(kn_{4}R_{4}\right)$$-h_{l}^{1}\left(kn_{5}R_{4}\right)$00000$n_{4}{j^{'}}_{l}\left(kn_{4}R_{4}\right)$$n_{4}{h^{'}}_{l}^{1}\left(kn_{4}R_{4}\right)$$-n_{5}{h^{'}}_{l}^{1}\left(kn_{5}R_{4}\right)$
while, for the TM modes, the even rows must be changed opportunely. In the Equation (3), *λ* is the resonance wavelength associated with the resonant wave vector *k* and *A* is the coefficients vector [25,40].

The system M × A = 0 has nontrivial solution only if the determinant of M is zero. For a given *λ* (for instance, the excitation wavelength of the laser source), the equation |det(M(*λ*,*l*))| = 0 represents the dispersion equation of the microbubble resonator and, hence, its zeros correspond to the eigenmodes of the same structure. The dispersion equation has to be solved in order to find the azimuthal mode number *l*, which must be chosen as a suitable natural number (integer). Using this requirement, the resonance wavelengths *λ_res_* of each WGMs can be determined for a given *l*. This can easily be extended to less or more layers, just by subtracting or adding extra rows with the proper input. The matrix will always be square because each additional layer adds two rows since the extra layer will be a layer in the region between the inner and the outer layer. In the case of N layers, this matrix has 2(N − 1) rows: two rows for each interface. On each row, only the entries corresponding to the layers next to the interface are nonzero. The solution of the system (Equation (3)) is not as simple as it seems. This is due to some numerical problems related to the bad initializing condition for the matrix related to the fact that some solutions are very close to each other. In order to avoid this problem, in this work we use a minimization approach looking for the minimum in the space for the values couple (*λ*,*l*) of the equation |det(M(*λ*,*l*))| = 0.

These values are then inserted in a singular value decomposition solver, developed in Matlab, in order to search the solution of the linear system, which can be solved up to a constant. The system solution is the null of the matrix M.

### 2.2. OMBR Sensitivity

When the internal biological layer (with a refractive index *n*_2_) undergoes an RI change due to some chemical/biochemical binding with molecules/analytes, a shift in the resonant wavelength would correspondingly take place. The sensitivity (*S*) of the wavelength shift versus the biological layer RI change can then be generally evaluated as *S* = ∂*λ*/∂*n*_2_. Nevertheless, it was theoretically demonstrated that, with a good approximation, this sensitivity value is strictly related to the percentage of the energy field inside the layer of interest [12,15,18]. In our case, this approximation leads to the following expression:

(4) $$\begin{array}{c} S=\frac{\partial \lambda }{\partial n_{2}}≅\frac{\lambda_{res}}{n_{eff}}\eta_{2}\\ \eta_{2}=\frac{I_{i}}{\sum_{j=1}^{4}I_{j}}\;i=1,\ldots ,\;5,\;for\;TE\;modes\\ \eta_{2}=\frac{n_{i}^{2}I_{i}}{\sum_{j=1}^{4}n_{j}^{2}I_{j}}\;i=1,\ldots ,\;5,\;for\;TM\;modes \end{array}$$

where $\lambda_{res}$ is the resonance wavelength, $n_{eff}=\lambda_{res}\;l/2\pi R$ is the effective refractive index and $\eta_{2}$ is the fraction of the field intensity in the biological layer. This situation can be reached when the peak of a WGM radial component of *n*-order presents a maximum in this region or, at least, the mode peak is located at the interface between the biological layer and the OMBR liquid core (the inner medium). In this way, a maximization of the evanescent field in the sensing region can be obtained with the resulting sensitivity increase of the device as reported in Equation (4). For this purpose, an optimization of the optical and geometrical parameters of the OMBR, the radial order of the excited WGM, the OMBR shell thickness, and the polymer layer thickness are discussed in the Section 3 for both kinds of structures under investigation. The sensitivity will be calculated both for TE and TM polarization, as reported in [12,15,18].

### 2.3. OMBR Limit of Detection 

The Limit of Detection (LoD), defined as the smallest detectable change of a physical quantity depends on the sensitivity *S* and the system resolution *R* as reported in the following [15]:

(5) $$D=\;\frac{R}{S}$$

where *R* is defined as 3-fold the standard deviation of the total sensor noise, which is related to the spectral noise, the amplitude noise, and the thermal noise, respectively, as is well explained in [15,40]. Hence, *R* is the minimum WGM wavelength shift detectable by the system, taking in account all the noise contributions mentioned above. Therefore, for our estimation we preferred to express the LoD as a function of sensitivity *S* and the minimally resolved spectral shift *δλ_m_* as reported in [18,41]:

(6) $$D=\;\frac{\delta \lambda_{m}}{S}$$

where *δλ_m_* is usually a fraction of the resonance linewidth *δλ* (from 1/10 to 1/100) which is in turn inversely proportional to the OMBR *Q*-factor:

(7) $$Q=\;\frac{\lambda }{\delta \lambda }$$

## 3. Results and Discussion 

### 3.1. Sensitivity for an OMBR without Inner Polymeric Coating

**Figure 3:**
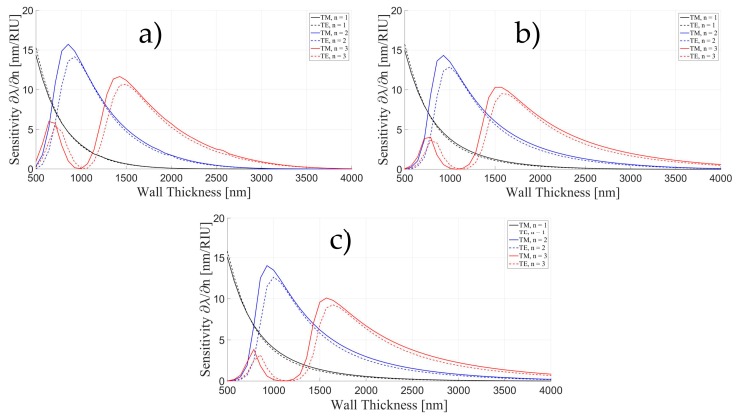
TE and TM mode sensitivity as a function of wall thickness for the first three radial orders and three different radii: (**a**) 50 μm; (**b**) 150 μm and (**c**) 250 μm.

We have calculated the sensitivity for the case of four layers (Figure 2a) for three different radii (50, 150, and 250 μm) varying the wall thicknesses (500 nm–4 μm). We have chosen the first three radial numbers, for both TE and TM polarizations. Figure 3 shows that there is no strong dependence on the radius of the resonator, even though the sensitivity decreased as the radius become larger. Another characteristic that can be observed is that TM mode has a higher sensitivity with its maximum slightly shifted to thinner walls. 

**Figure 4:**
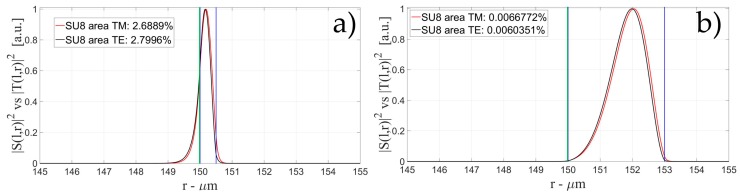
Electric field distribution of WGMs along the radial direction for TE (|*S*(*l,r*)*|*^2^) and TM (|*T*(*l,r*)*|*^2^) modes for the 1st radial order: (**a**) for a wall of about 500 nm (maximum sensitivity); and (**b**) for a wall of about 3 μm (minimum sensitivity). Legends show the fraction of |*S*(*l,r*)*|*^2^ and |*T*(*l,r*)*|*^2^ inside the biological layer.

**Figure 5:**
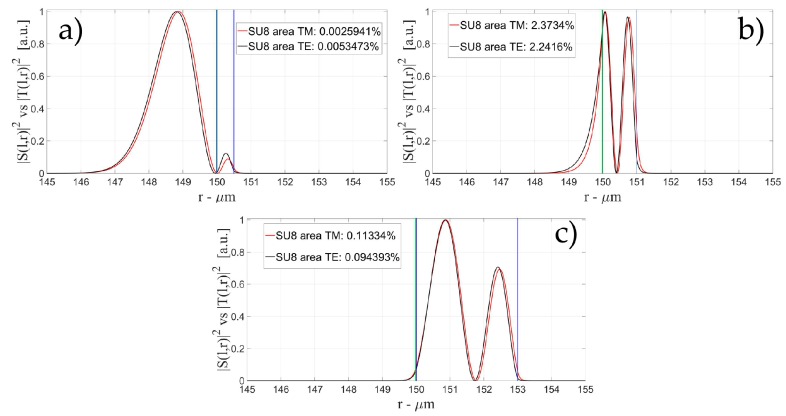
Electric field distribution of WGMs along the radial direction for TE (|*S*(*l,r*)*|*^2^) and TM (|*T*(*l,r*)*|*^2^) modes for the 2nd radial order and wall thickness: (**a**) for a wall of about 500 nm (minimum sensitivity); (**b**) for a wall of about 1 μm (maximum sensitivity); and (**c**) for a wall of about 3 μm (minimum sensitivity). Legends show the fraction of |*S*(*l,r*)*|*^2^ and |*T*(*l,r*)*|*^2^ inside the biological layer.

**Figure 6:**
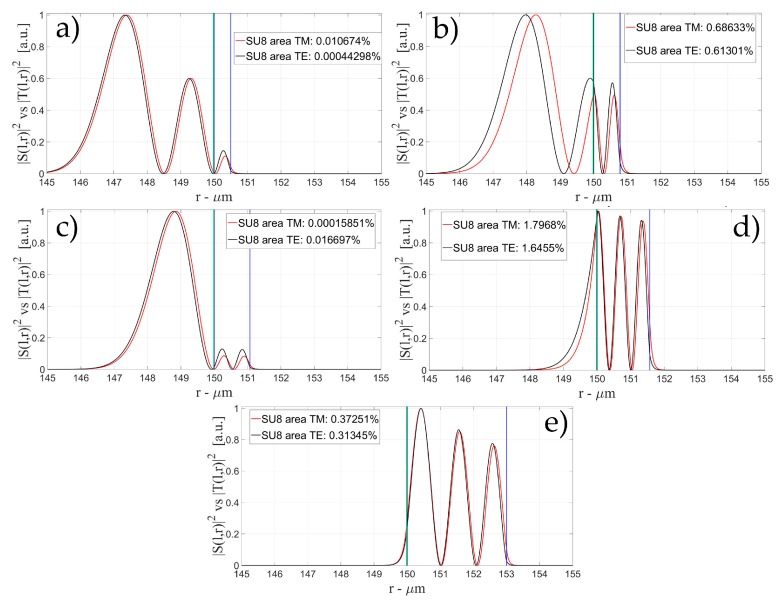
Electric field distribution of WGMs along the radial direction for TE (|*S*(*l,r*)*|*^2^) and TM (|*T*(*l,r*)*|*^2^) modes for the 2nd radial order and wall thickness: (**a**) for a wall of about 500 nm (minimum sensitivity); (**b**) for a wall of about 800 nm (maximum sensitivity); (**c**) for a wall of about 1 μm (minimum sensitivity); (**d**) for a wall of about 1.6 μm (maximum sensitivity); and (**e**) for a wall of about 3 μm (minimum sensitivity). Legends show the fraction of |*S*(*l,r*)*|*^2^ and |*T*(*l,r*)*|*^2^ inside the biological layer.

Figure 4, Figure 5 and Figure 6 show the intensity distribution versus the radial position for TE (|*S*(*l,r*)*|*^2^) and TM (|*T*(*l,r*)*|*^2^) polarization for the first three modes, respectively, for a radius of 150 μm. We have chosen to plot the intensity radial distribution for the maxima and minima of the sensitivity for each radial order. Figure 4a shows the intensity distribution for a wall of about 500 nm thick (maximum); as can be seen, the 1st order radial mode has a good penetration inside the OMBR but it has the entire field inside the silica shell for a wall of about 3 μm (almost zero sensitivity). Figure 5 shows the intensity distribution for the 2nd order radial mode whereas Figure 6 shows the intensity distribution for the 3rd order radial mode.

Figure 5a shows the intensity distribution of the 2nd radial order mode for a wall of about 500 nm (1st minimum of sensitivity) with no field at the interface, Figure 5b shows the distribution for a wall of about 1 μm (maximum of sensitivity) with a good evanescent field at the interface, and Figure 5c shows the distribution for a wall of about 3 μm (2nd minimum of sensitivity) with a very small evanescent tail. 

Figure 6a shows the intensity distribution of the 3rd radial order mode for a wall of about 500 nm (1st minimum of sensitivity) with no field at the interface (similar to Figure 6c, with a wall thickness of about 1 μm), Figure 6b shows the distribution for a wall of about 800 nm (1st maximum of sensitivity) with a good evanescent field at the interface, Figure 6d shows the distribution for a wall of about 1.6 μm (1st minimum of sensitivity) with a good evanescent tail penetrating in the OMBR, and Figure 6e shows the distribution for a wall of about 3 μm (3rd minimum of sensitivity) with a very small evanescent tail.

### 3.2. Sensitivity for an OMBR with an Inner Polymeric Coating

In this case, we calculated the sensitivity of an OMBR with polymeric layer with a variable thickness on its inner surface between the glass wall and the bio-layer (see Figure 2b). The chosen polymer is SU-8, with a refractive index of about 1.582 at a wavelength of 780 nm. The simulations have been performed for a fixed radius of the OMBR, namely, 150 μm, which is quite close to the standard radius of our homemade OMBR. We have calculated the sensitivity for several polymer thicknesses: 50, 100, 200, 400, 800, 1000, 1200, 1400 and 1600 nm as a function of wall thickness, radial order, TE and TM polarizations, for a radius of 150 μm.

**Figure 7:**
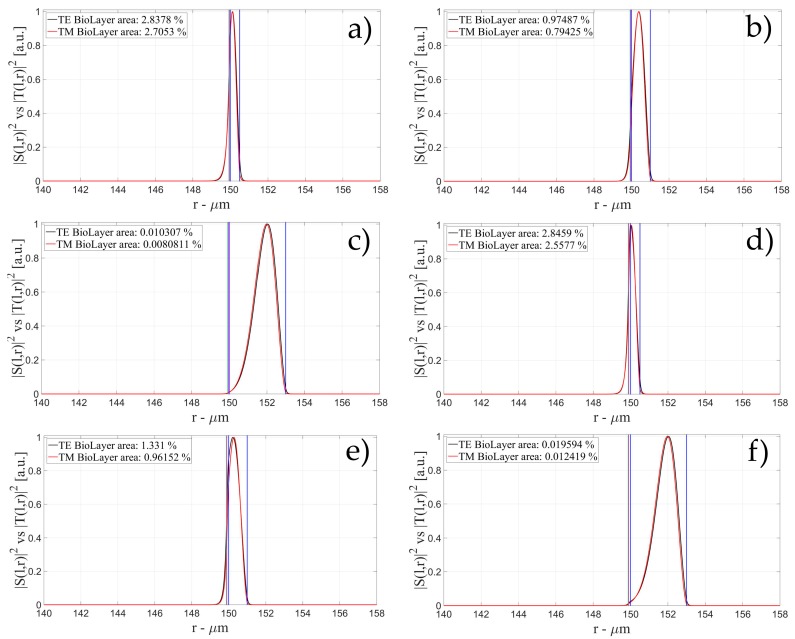
Electric field distribution of WGMs along the radial direction for TE (|*S*(*l,r*)*|*^2^) and TM (|*T*(*l,r*)*|*^2^) modes for the 1st radial order: (**a**) for a wall of about 500 nm and a polymer thickness of about 50 nm; (**b**) for a wall of about 1 μm and a polymer thickness of about 50 nm and (**c**) for a wall of about 3 μm and a polymer thickness of about 50 nm; (**d**) for a wall of about 500 nm and a polymer thickness of about 100 nm; (**e**) for a wall of about 1 μm and a polymer thickness of about 100 nm and (**f**) for a wall of about 3 μm and a polymer thickness of about 100 nm. Legends show the fraction of |*S*(*l,r*)*|*^2^ and |*T*(*l,r*)*|*^2^ inside the biological layer.

**Figure 8:**
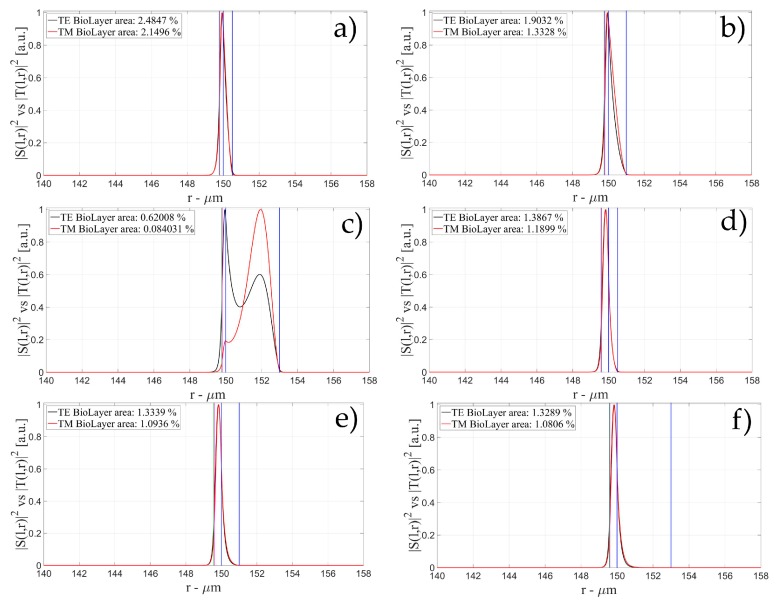
Electric field distribution of WGMs along the radial direction for TE (|*S*(*l,r*)*|*^2^) and TM (|*T*(*l,r*)*|*^2^) modes for the 1st radial order: (**a**) for a wall of about 500 nm and a polymer thickness of about 200 nm; (**b**) for a wall of about 1 μm and a polymer thickness of about 200 nm and (**c**) for a wall of about 3 μm and a polymer thickness of about 200 nm; (**d**) for a wall of about 500 nm and a polymer thickness of about 400 nm; (**e**) for a wall of about 1 μm and a polymer thickness of about 400 nm and (**f**) for a wall of about 3 μm and a polymer thickness of about 400 nm. Legends show the fraction of |*S*(*l,r*)*|*^2^ and |*T*(*l,r*)*|*^2^ inside the biological layer.

**Figure 9:**
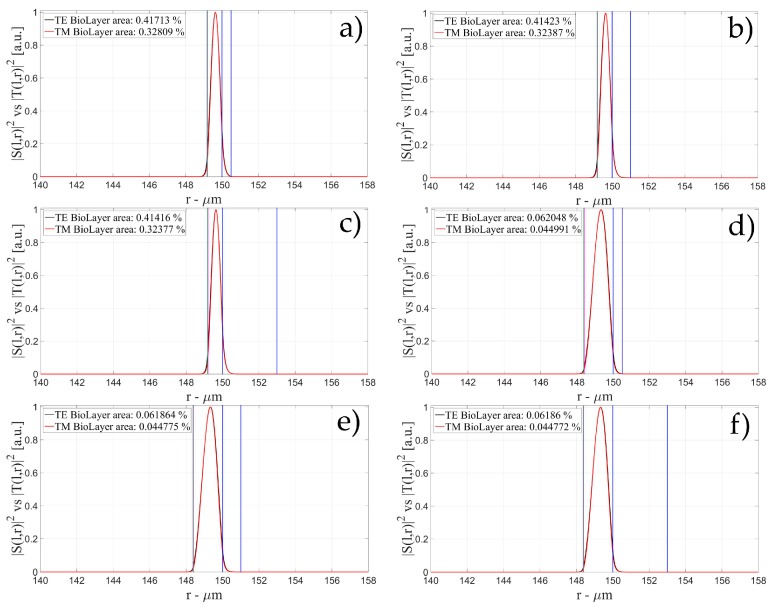
Electric field distribution of WGMs along the radial direction for TE (|*S*(*l,r*)*|*^2^) and TM (|*T*(*l,r*)*|*^2^) modes for the 1st radial order: (**a**) for a wall of about 500 nm and a polymer thickness of about 800 nm; (**b**) for a wall of about 1 μm and a polymer thickness of about 800 nm and (**c**) for a wall of about 3 μm and a polymer thickness of about 800 nm; (**d**) for a wall of about 500 nm and a polymer thickness of about 1600 nm; (**e**) for a wall of about 1 μm and a polymer thickness of about 1600 nm and (**f**) for a wall of about 3 μm and a polymer thickness of about 1600 nm. Legends show the fraction of |*S*(*l,r*)*|*^2^ and |*T*(*l,r*)*|*^2^ inside the biological layer.

**Figure 10:**
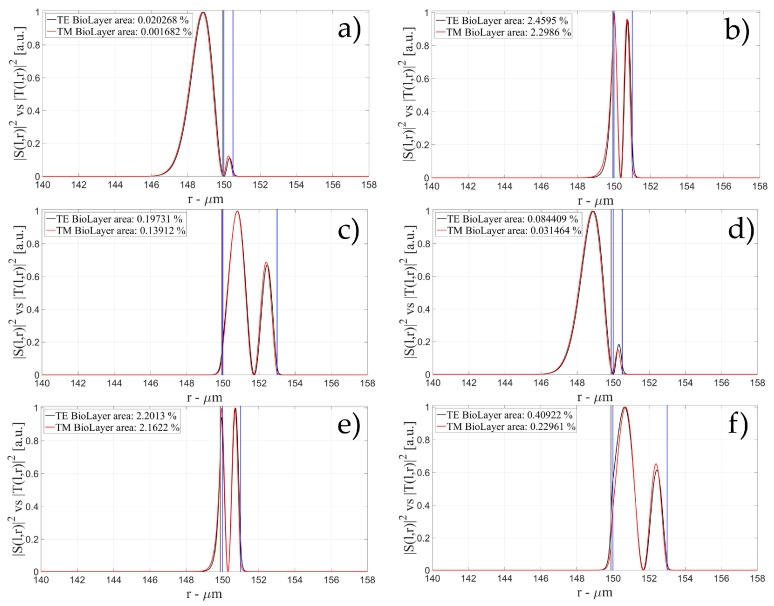
Electric field distribution of WGMs along the radial direction for TE (|*S*(*l,r*)*|*^2^) and TM (|*T*(*l,r*)*|*^2^) modes for the 2nd radial order: (**a**) for a wall of about 500 nm and a polymer thickness of about 50 nm; (**b**) for a wall of about 1 μm and a polymer thickness of about 50 nm and (**c**) for a wall of about 3 μm and a polymer thickness of about 50 nm; (**d**) for a wall of about 500 nm and a polymer thickness of about 100 nm; (**e**) for a wall of about 1 μm and a polymer thickness of about 100 nm and (**f**) for a wall of about 3 μm and a polymer thickness of about 100 nmLegends show the fraction of |*S*(*l,r*)*|*^2^ and |*T*(*l,r*)*|*^2^ inside the biological layer.

**Figure 11:**
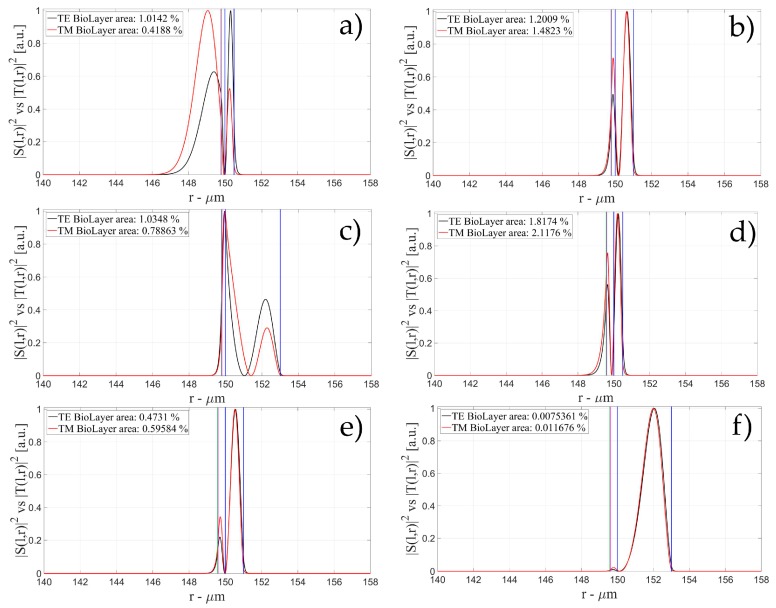
Electric field distribution of WGMs along the radial direction for TE (|*S*(*l,r*)*|*^2^) and TM (|*T*(*l,r*)*|*^2^) modes for the 2nd radial order: (**a**) for a wall of about 500 nm and a polymer thickness of about 200 nm; (**b**) for a wall of about 1 μm and a polymer thickness of about 200 nm and (**c**) for a wall of about 3 μm and a polymer thickness of about 200 nm; (**d**) for a wall of about 500 nm and a polymer thickness of about 400 nm; (**e**) for a wall of about 1 μm and a polymer thickness of about 400 nm and (**f**) for a wall of about 3 μm and a polymer thickness of about 400 nm Legends show the fraction of |*S*(*l,r*)*|*^2^ and |*T*(*l,r*)*|*^2^ inside the biological layer.

**Figure 12:**
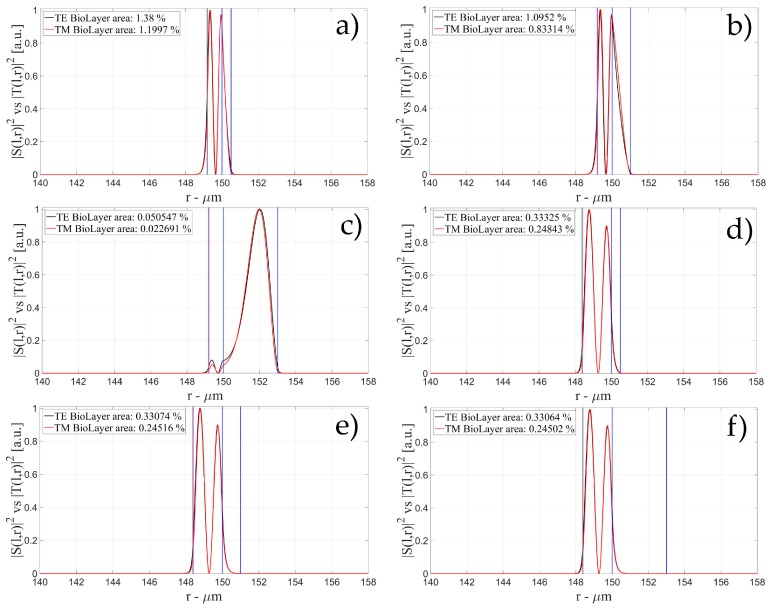
Electric field distribution of WGMs along the radial direction for TE (|*S*(*l,r*)*|*^2^) and TM (|*T*(*l,r*)*|*^2^) modes for the 2nd radial order: (**a**) for a wall of about 500 nm and a polymer thickness of about 800 nm; (**b**) for a wall of about 1 μm and a polymer thickness of about 800 nm and (**c**) for a wall of about 3 μm and a polymer thickness of about 800 nm; (**d**) for a wall of about 500 nm and a polymer thickness of about 1600 nm; (**e**) for a wall of about 1 μm and a polymer thickness of about 1600 nm and (**f**) for a wall of about 3 μm and a polymer thickness of about 1600 nm. Legends show the fraction of |*S*(*l,r*)*|*^2^ and |*T*(*l,r*)*|*^2^ inside the biological layer.

**Figure 13:**
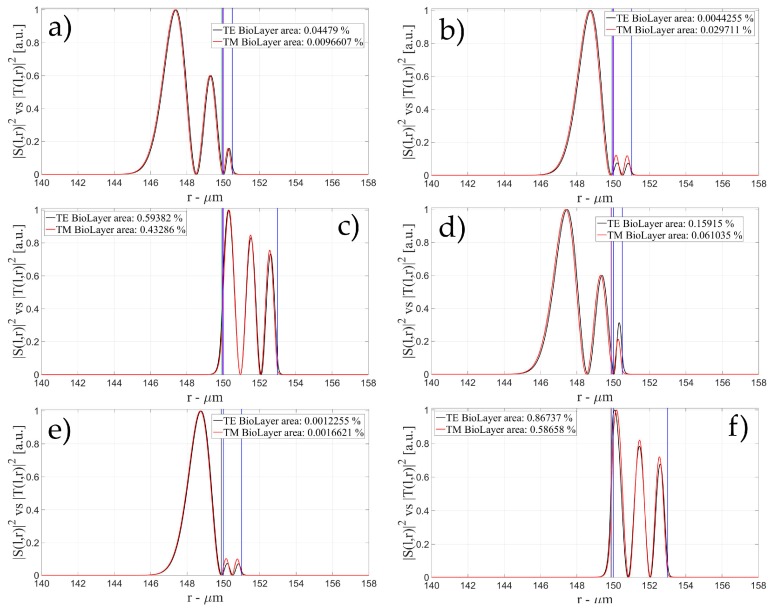
Electric field distribution of WGMs along the radial direction for TE (|*S*(*l,r*)*|*^2^) and TM (|*T*(*l,r*)*|*^2^) modes for the 3rd radial order: (**a**) for a wall of about 500 nm and a polymer thickness of about 50 nm; (**b**) for a wall of about 1 μm and a polymer thickness of about 50 nm and (**c**) for a wall of about 3 μm and a polymer thickness of about 50 nm; (**d**) for a wall of about 500 nm and a polymer thickness of about 100 nm; (**e**) for a wall of about 1 μm and a polymer thickness of about 100 nm and (**f**) for a wall of about 3 μm and a polymer thickness of about 100 nm. Legends show the fraction of |*S*(*l,r*)*|*^2^ and |*T*(*l,r*)*|*^2^ inside the biological layer.

**Figure 14:**
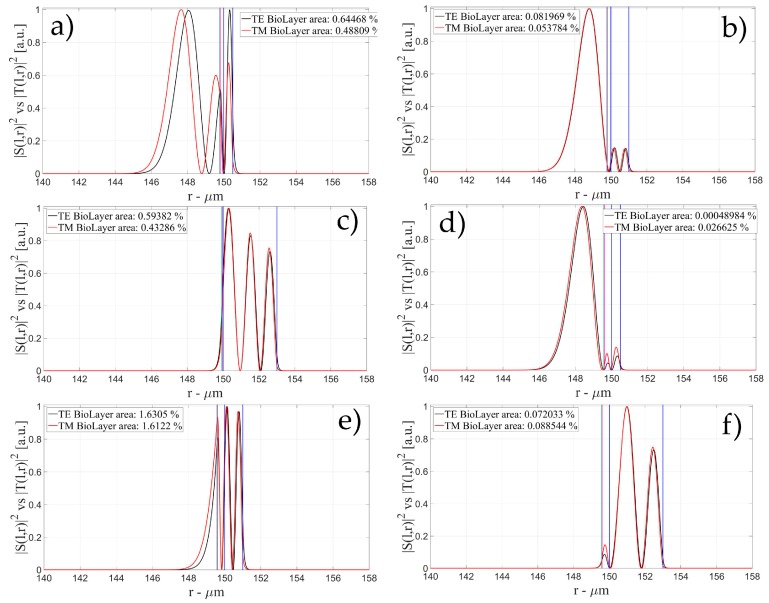
Electric field distribution of WGMs along the radial direction for TE (|*S*(*l,r*)*|*^2^) and TM (|*T*(*l,r*)*|*^2^) modes for the 3rd radial order: (**a**) for a wall of about 500 nm and a polymer thickness of about 200 nm; (**b**) for a wall of about 1 μm and a polymer thickness of about 200 nm and (**c**) for a wall of about 3 μm and a polymer thickness of about 200 nm; (**d**) for a wall of about 500 nm and a polymer thickness of about 400 nm; (**e**) for a wall of about 1 μm and a polymer thickness of about 400 nm and (**f**) for a wall of about 3 μm and a polymer thickness of about 400 nm Legends show the fraction of |*S*(*l,r*)*|*^2^ and |*T*(*l,r*)*|*^2^ inside the biological layer.

**Figure 15:**
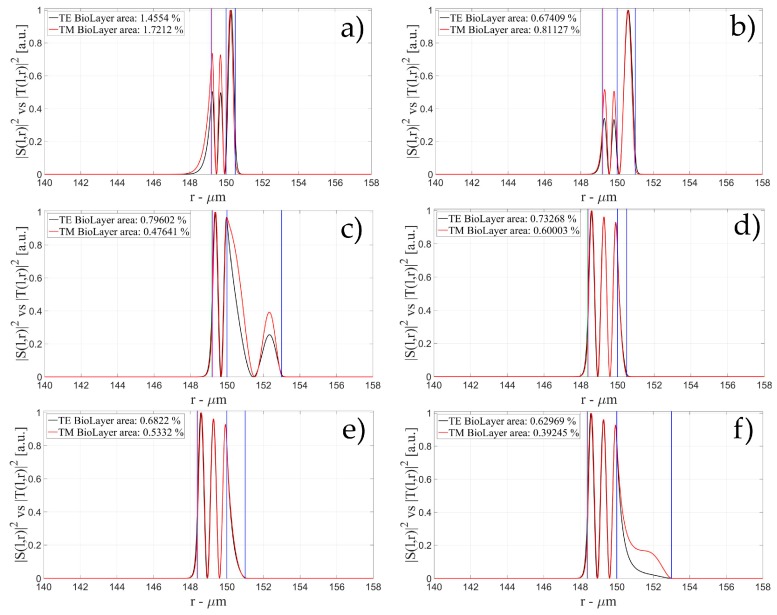
Electric field distribution of WGMs along the radial direction for TE (|*S*(*l,r*)*|*^2^) and TM (|*T*(*l,r*)*|*^2^) modes for the 3rd radial order: (**a**) for a wall of about 500 nm and a polymer thickness of about 800 nm; (**b**) for a wall of about 1 μm and a polymer thickness of about 800 nm and (**c**) for a wall of about 3 μm and a polymer thickness of about 800 nm; (**d**) for a wall of about 500 nm and a polymer thickness of about 1600 nm; (**e**) for a wall of about 1 μm and a polymer thickness of about 1600 nm and (**f**) for a wall of about 3 μm and a polymer thickness of about 1600 nm Legends show the fraction of |*S*(*l,r*)*|*^2^ and |*T*(*l,r*)*|*^2^ inside the biological layer.

The characteristics of the biological layer have been kept equal to the previous case. Figure 7, Figure 8, Figure 9, Figure 10, Figure 11, Figure 12, Figure 13, Figure 14 and Figure 15 show the intensity distribution versus the radial position for TE (|*S*(*l,r*)*|*^2^) and TM (|*T*(*l,r*)*|*^2^) polarization for the first three modes, respectively, for a radius of 150 μm. These panels show the intensity distribution for the polymer thicknesses of 50, 100, 200, 400, 800, and 1600 nm, each of them for three different wall thicknesses, for an easy comparison with the four-layer case.

**Figure 16:**
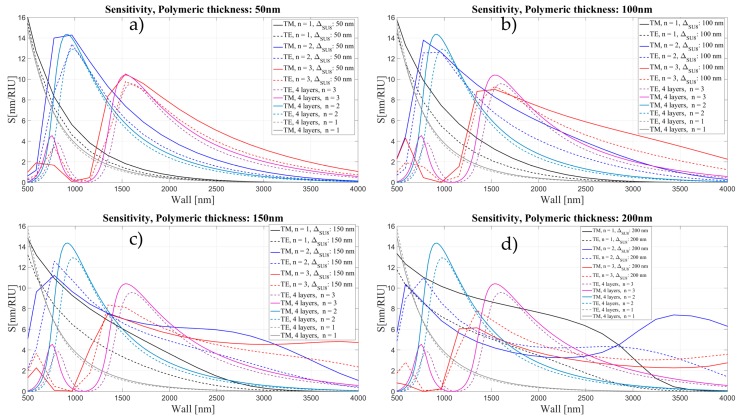
Sensitivity (nm/RIU) for the 4 and 5 layers model as a function of wall thickness for two different polarizations and 3 mode orders, corresponding to different thickness of polymeric layer: (**a**) 50 nm; (**b**) 100 nm; (**c**) 150 nm; and (**d**) 200 nm.

In Figure 16, we show the calculated sensitivity for the OMBR with and without the inner polymeric layer, as a function of wall thickness for two different polarizations and three mode orders. 

When the polymer layer is thin, the sensitivity in the biological layer shows a similar behavior to the sensitivity without the polymer. For higher order modes, however, the sensitivity is slightly higher when the inner wall of the OMBR is coated with SU-8. In this case, the light is still confined in the OMBR wall. In Figure 16d, it can be seen that the 2nd radial order mode shows a second maximum at 3.5 μm wall thickness and 200 nm polymer thickness.

**Figure 17:**
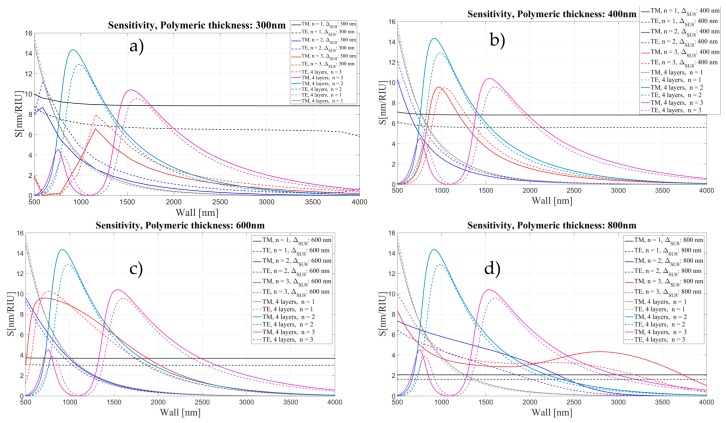
Sensitivity (nm/RIU) for the four- and five-layer models as a function of wall thickness for two different polarizations and three mode orders, corresponding to different thickness of polymeric layer: (**a**) 300 nm; (**b**) 400 nm; (**c**) 600 nm; and (**d**) 800 nm.

**Figure 18:**
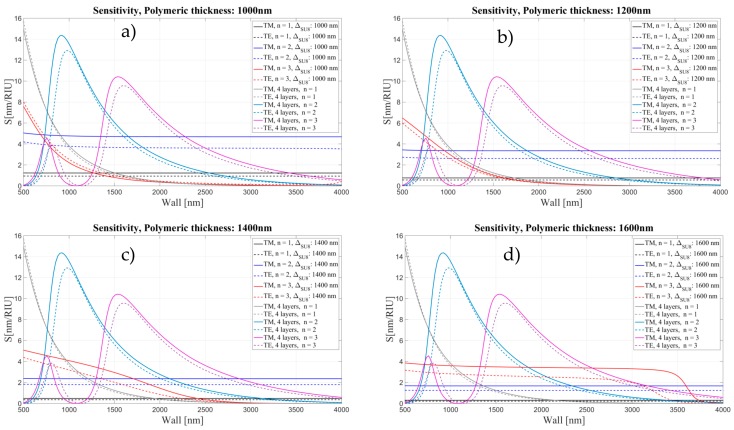
Sensitivity (nm/RIU) for the four- and five-layer models as a function of wall thickness for two different polarizations and three mode orders, corresponding to different thickness of polymeric layer: (**a**) 1000 nm; (**b**) 1200 nm; (**c**) 1400 nm; and (**d**) 1600 nm.

For thick polymeric layers (t ≥ 300 nm), the sensitivity loses it characteristic oscillatory behavior and it becomes flat, almost constant for all wall thicknesses (see Figure 17 and Figure 18). First the maximum of the sensitivity for each order mode shifts to thinner walls and with a further increase of the polymeric layer it becomes flat for each order mode.

Having a constant sensitivity can be very useful in terms of fabrication method and performances, the sensitivity becomes independent of the wall thickness and OMBR could be more robust and easy to manipulate. For very thick layers and thicker walls, coupling the light inside the polymer layer could be problematic due to the lack of evanescent field outside the external wall of the OMBR, where evanescent coupling with the bus waveguide takes place.

For very thick layers, the sensitivity drops to very low values, close to zero. In this other case, the light is totally confined inside the polymer. This is equivalent to creating a polymeric OMBR inside the silica one. 

Depositing 300 nm polymeric layers inside the silica OMBR is good compromise for having a constant RI sensitivity and a robust device, avoiding the mentioned coupling problems.

## 4. Limit of Detection

Considering a *Q* factor of about 10^6^ and a working operation wavelength of 770 nm, we have a resonance width *δλ* of about 0.77 pm. 

If system noises are reduced and controlled, the resolution of our system can be estimated as fraction (e.g., 1/20) of *δλ*, then:

{LoD= δλmS(nmRIU)≅120δλS(nmRIU)=120λQ1SQ=λδλ

**Figure 19:**
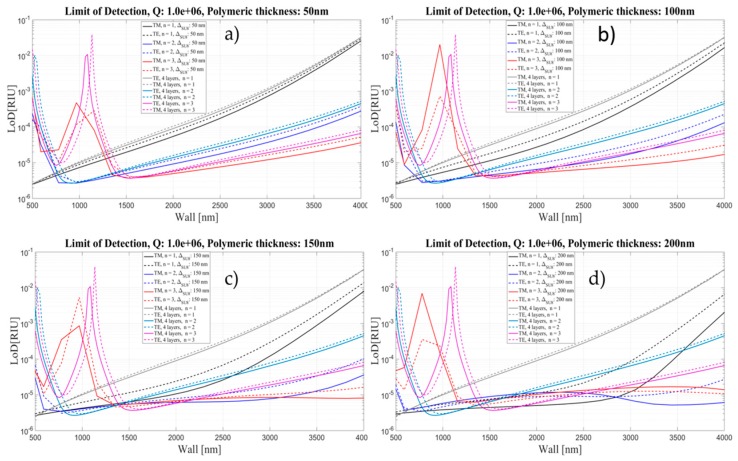
Limit of Detection for the four- and five-layer models as a function of Wall thickness for two different polarizations and three mode orders, corresponding to different thickness of polymeric layer: (**a**) 50 nm; (**b**) 100 nm; (**c**) 150 nm; and (**d**) 200 nm. *Q* is assumed to be about 10^6^.

**Figure 20:**
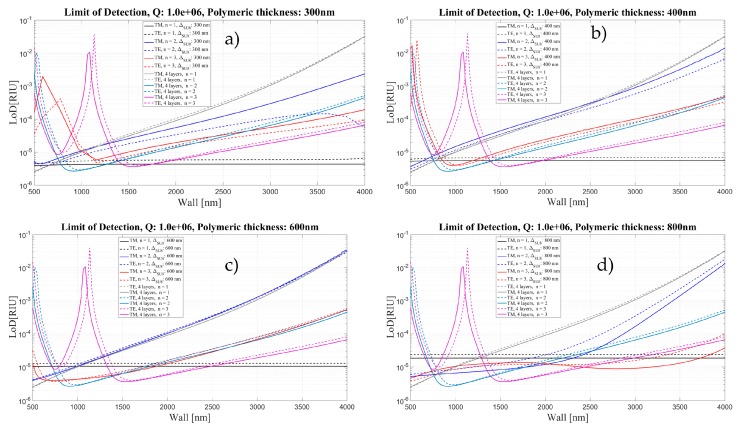
Limit of Detection for the four- and five-layer models as a function of Wall thickness for two different polarizations and three mode orders, corresponding to different thickness of polymeric layer: (**a**) 300 nm; (**b**) 400 nm; (**c**) 600 nm; and (**d**) 800 nm. *Q* is assumed to be about 10^6^.

**Figure 21:**
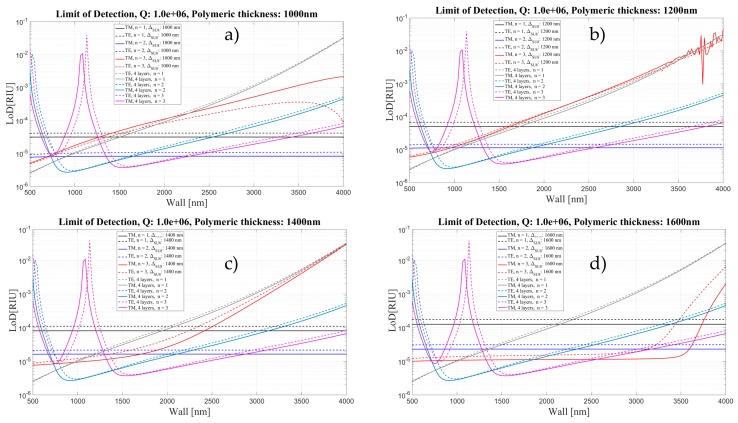
Limit of Detection for the four- and five-layer models as a function of Wall thickness for two different polarizations and three mode orders, corresponding to different thickness of polymeric layer: (**a**) 1000 nm; (**b**) 1200 nm; (**c**) 1400 nm; and (**d**) 1600 nm. *Q* is assumed to be about 10^6^.

Figure 19, Figure 20 and Figure 21 show a comparison of the limit of detection for both cases, without and with an internal polymeric layer as a function of wall thickness for two different polarizations and three mode orders. Each panel corresponds to different thickness of polymeric layer, ranging from 50 to 1600 nm. 

The LoD behavior follows the behavior of the sensitivity.

The results shown here confirm that depositing 300 nm polymeric layers inside the silica OMBR is a good compromise for having a constant LoD of about 4.2 × 10^−6^ for the TM mode and 5.4 × 10^−6^ for the TE mode. Future work will be developed in order to implement experimentally the theoretical results presented here. We are adapting the well-developed coating procedures used in gas chromatography columns as in [42]. Preliminary results showed that we are able to coat capillaries with inner polymeric layers ranging from 10 nm to several microns [43].

## 5. Conclusions

We have calculated the sensitivity and LoD of an OMBR-based sensor using a transfer matrix approach. As expected, the OMBR sensitivity and LoD depends on the wall thickness and the radial order mode excited in the OMBR. The higher is the mode, the thicker can be the OMBR wall. Though the polymer layer does not improve the sensitivity, it relaxes the fabrication constraints and allows the use of more robust OMBR.
